# Human-animal contact and zoonotic exposure from wild and domestic animals: A cross-sectional study in wildlife-rich areas of Bolivia, Chile, and Guatemala

**DOI:** 10.1016/j.onehlt.2026.101438

**Published:** 2026-05-08

**Authors:** Caroline Kuhn, Katja Radon, Fabiana Marcela Pérez Morales, Marcia Adler, Carlos Fernando Pinto Navia, María Soledad Burrone, Carlos Roberto Vásquez-Almazán, Denise Siqueira de Carvalho, María Teresa Solis Soto, on behalf of the KAP wildlife study group

**Affiliations:** a Center for International Health, Ludwig-Maximilian-University Hospital, 80336 Munich, Germany; b CIHLMU OH TARGET Competence Center, Universidad San Francisco Xavier de Chuquisaca, Sucre, Bolivia; c Institute of Health Sciences, Universidad de O'Higgins, Rancagua, Chile; d Museo de Historia Natural - Escuela de Biología, Universidad San Carlos de Guatemala, Guatemala City, Guatemala; e Federal University of Paraná, Curitiba, Brazil

**Keywords:** Zoonoses, One health, Disease transmission, infectious, Health knowledge, attitudes, practice, Risk factors, Human-animal interaction, Latin America

## Abstract

**Introduction:**

Human–animal contact (HAC) is a key driver of zoonotic disease emergence and transmission, yet population-based data from Latin America remain limited. The objective of this study was to characterize HAC patterns and estimate zoonotic exposure in wildlife-rich settings across Bolivia, Chile, and Guatemala.

**Methods:**

A cross-sectional questionnaire was administered to 2389 participants in rural, urban, and protected areas to assess direct, indirect, and foodborne contact with domestic and wild animals. Patterns of HAC were visualized using heatmaps, and a proxy-based exposure index was developed based on literature-derived weightings for rabies, brucellosis, and bovine tuberculosis. Associations with sociodemographic and knowledge-related factors were analyzed using multivariable regression models.

**Results:**

Overall, 92% of participants reported domestic animal contact—most frequently with dogs, cats, poultry, and swine—while 32% reported wild animal contact, predominantly with wild birds, rodents, and bats. 99% of individuals with wildlife contact also reported contact with domestic animals. Direct interactions, including ownership, handling, and slaughtering, were most common, whereas indirect and foodborne contacts were less frequent. Adolescents and participants with lower income and education showed higher exposure scores. However, in adjusted models, country and area of residence were the most consistent determinants of exposure. Rural residence was associated with greater exposure to brucellosis and bovine tuberculosis, and prior zoonosis training with lower exposure to rabies.

**Conclusion:**

The study highlights the co-occurrence of wildlife and domestic human-animal contacts. Further research integrating HAC, pathogen surveillance, and host vulnerability with a One Health approach is critical for identifying high-risk human-animal-environment interfaces.

## Highlights

• Wildlife and domestic human-animal contact frequently co-occur.
• Direct interactions, including ownership, handling, and slaughtering, were common.
• The highest estimated zoonotic exposure levels were observed in Bolivia.
• Zoonotic risk integrates exposure, pathogen surveillance, and host vulnerability.

## 1 Introduction

Zoonotic diseases, transmitted between animals and humans, represent an increasing global health concern, highlighting the need for coordinated One Health strategies that link human, animal, and environmental health. Emerging zoonotic diseases are defined as an increase in incidence or geographic range, movement into new host populations, recent discovery, or causation by a newly evolving pathogen [1]. While 72% of such disease events originate from wildlife [2], livestock and companion animals also contribute significantly to transmission [3], [4], [5]. A recent example is the spillover of the highly pathogenic avian influenza H5N1 virus to dairy cattle in 2024 [6].

In contrast, endemic zoonotic diseases persist in specific geographic locations [7] and disproportionately affect impoverished populations in Low- and Middle-Income Countries [8]. Prioritization efforts in Latin America have recognized both types of threats: in 2015, local ministries of health and agriculture identified rabies, leptospirosis, brucellosis, tuberculosis, and salmonellosis as the top five endemic zoonotic diseases, while the top five prioritized emerging zoonotic diseases were avian influenza, Ebola viral disease, bovine spongiform encephalopathy, chikungunya, and West Nile virus [9]. Domestic animals play a central role as hosts of endemic zoonotic diseases [10], and many of these pathogens circulate across both wild and domestic species, emphasizing the importance of cross-species dynamics at the human–animal interface.

In this context, human-animal contacts (HAC) – which are defined as any interaction between humans and animals – pose the risk for pathogen transmission. Zoonotic pathogens are passed on via direct contact (e.g., animal bites), indirect contact (e.g., airborne or waterborne transmission pathways), foodborne transmission, and vector-borne transmission [11]. The characteristics and frequency of these HAC have been demonstrated to influence the prevalence of emerging and endemic zoonotic diseases [12], [13]. For example, frequent direct contact between humans and animals increases the probability of pathogens crossing species boundaries, thereby fostering the emergence of novel pathogens [14].

Compared to other continents, research in zoonotic diseases in Latin America remains limited and often outdated [15]. While countries such as Chile have relatively well-established surveillance systems [16], others, including Bolivia and Guatemala, face important gaps in research and monitoring for zoonoses [17], [18], [19], [20]. Although Latin America performs comparatively well in One Health-oriented interventions, it ranks lower in conventional approaches targeting specific transmission routes [21].

Contemporary strategies for addressing zoonotic disease risks increasingly prioritize primary prevention over reactive measures. This shift is grounded in the recognition that the root cause of zoonotic pandemics lies in the initial pathogen transmission from animals to humans and the finding that preventive measures are 20 times more cost-effective [22]. This suggests a critical need to improve our understanding of zoonotic exposure and transmission pathways. A promising framework for quantifying zoonotic risk is to assess human exposure indicators, which integrate two critical dimensions: (1) Pathogen circulation within relevant domestic and wild animal populations, and (2) contact intensity between humans and animals [23].

Little is known about HAC patterns in the context of zoonotic transmission pathways in domestic and wild animals [4]. Therefore, the objective of this study is to characterize patterns of HAC across wildlife-rich urban and rural populations in Bolivia, Chile, and Guatemala, quantify potential zoonotic exposure based on direct, indirect, and foodborne pathways, and identify high-risky practices at the human-animal interface regarding rabies, brucellosis and bovine tuberculosis, three of the top five prioritized endemic zoonotic diseases in Latin America.

## 2 Methods

### 2.1 Study area

**Fig. 1:**
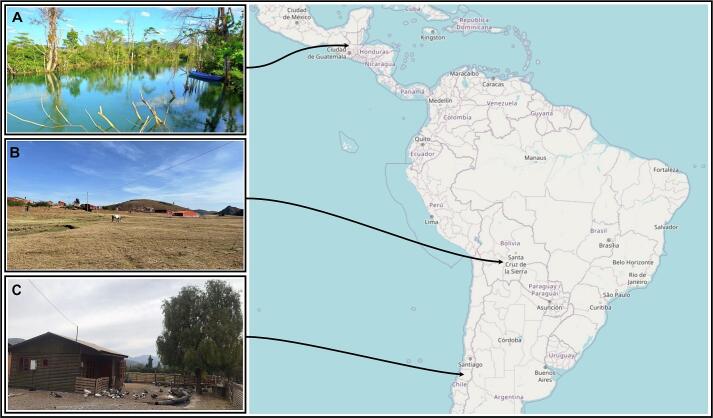
Study areas from North to South. A) Ecoregion Lachua, Guatemala; B) Presto, Province of Jaime Zudañez, Bolivia; C) Coya Chacayes, O'Higgins Region, Chile. The map was exported from openstreetmap.org and photos (A: Carlos Roberto Vásquez-Almazán; B: Caroline Kuhn; C: Pilar Macarena Mansilla Vivar) were reused with permission.

A cross-sectional study was conducted between September, 152022 and September 30, 2024, in Bolivia, Chile and Guatemala. In each country a geographically distinct area was selected, representing rural and urban population in or close to protected areas to capture biodiverse and wildlife-rich areas with diverse cultural contexts across Latin America (Fig. 1).

**In Guatemala**, the study was implemented in the Lachua Ecoregion, located around Laguna Lachua National park, a tropical wetland region with approximately 12,500 inhabitants and one of the country's most biodiverse zones. It hosts 55 indigenous communities primarily composed of the Maya-Q'eqchi´ people, who rely on agriculture and sustainable use of wildlife and forest products.

**In Bolivia**, the study took place in the Department of Chuquisaca, Province of Jaime Zudañez, Municipality of Presto. This municipality covers 1444 km^2^ and has a population of 12,385. It comprises 33 communities and two neighborhood councils, with a predominantly Quechua-speaking population. The area lies within the El Palmar Integrated Management Natural Area (595 km^2^), a protected zone emphasizing biodiversity conservation and cultural heritage preservation in inter-Andean mesothermal semi-arid valleys.

**In Chile**, the research was carried out in the O'Higgins Region, centrally located and home to 914,555 inhabitants. Study areas included the municipalities of Navidad, Doñihue (Lo Miranda), and Machalí (Coya), spanning varied topographies and a Mediterranean climate. Coastal regions are humid and temperate, while mountainous zones experience sub-zero temperatures in winter. Environmental challenges in this area include livestock-wildlife conflict and recurrent droughts.

### 2.2 Study population

A two-stage cluster sampling method was employed in each country targeting to recruit a total of 900 participants. This involved randomly selecting Enumeration Areas to include 300 individuals each from rural, urban, and protected areas. All household members aged 10 years and older were invited to participate. The sample size considered the balance between feasibility of implementing fieldwork, and statistical robustness, aiming for 100 people in each of the three age categories—adolescent, adults, and the elderly—per area. Prior to the fieldwork, the study was socialized with local authorities and local media to inform about the study and encourage participation. Community representatives helped facilitate access to the target population. Written informed consent was collected from each participant or their legal guardian before data collection began. The final study sample comprised 2389 participants, representing those individuals who were successfully reached during fieldwork.

### 2.3 Materials

A standardized questionnaire (Supporting Information, File 1) was developed based on established knowledge, attitudes, and practices (KAP) tools [24], [25], and included items on socio-demographic characteristics, education, occupation, and zoonotic disease-related KAP. The tool was translated into the local languages and underwent pilot testing in each country to ensure comprehensibility and cultural appropriateness. Feedback from the pilots was used to refine the instrument. Trained interviewers, fluent in the local languages, conducted face-to-face interviews. Responses were standardized and recorded using Epi Info™ or the Open Data Kit platform for data entry and management.

### 2.4 Variable definition

The variables for the analysis were defined as follows:

Sociodemographic variables

• Age Group: Participants were categorized into three age groups based on their reported age: Adolescents (10–17 years), Adults (18–60 years), and Elderly (>60 years).
• Gender: Self-reported gender was recorded as Female, Male, or Other.
• Education: Educational attainment was classified as None, Primary (completion of primary education), Secondary (completion of high school or equivalent), and Tertiary (college, university, or professional training). For participants under 18, parental education level was used.
• Monthly Income: Income was reported in country-specific currency and categorized relative to the national minimum wage (NMW): < 0.5 NMW, 0.5–1 NMW, 1–2 NMW, > 2 NMW.
• Country: Bolivia, Chile, or Guatemala.
• Area: Urban, Rural, or Protected Area

Knowledge-associated variables

• Previous zoonosis training: Participants indicated whether they had ever received training related to zoonotic diseases, wildlife trade, or wildlife consumption (Yes/No)

Human-animal contact was assessed through self-reported practices over the preceding 12 months, grouped into three potential transmission routes: direct, indirect, and foodborne. Direct contact included physical interactions with animals, such as handling, bites, slaughtering, or keeping animals. Indirect contact referred to environmental or proximity-based exposures, such as animals entering the household, contact with animal feces, or shared water sources. Foodborne contact included the consumption of animal products, particularly raw or undercooked meat. Each of these variables was recorded as binary (yes/no) based on the participant's self-report.

### 2.5 Zoonotic exposure index for human-animal contacts

The questionnaire was originally developed within a broader KAP framework regarding the risk of zoonotic diseases, wildlife trade, and wildlife consumption. As participants frequently reported contact with both wildlife and domestic animals, the human–wildlife–livestock interface emerged as an important context for zoonotic exposure. For this analysis, selected practice-related variables were therefore re-conceptualized as indicators of zoonotic exposure. To summarize self-reported human–animal contact patterns in relation to zoonotic exposure pathways, we developed a literature-informed zoonotic exposure index (Table 1). The index was designed as a proxy measure of potential exposure and not as a measure of infection risk. We focused on rabies, brucellosis, and bovine tuberculosis because these zoonoses were identified among the priority endemic zoonotic diseases in Latin America [9]. A weighted scoring system (0–3) was assigned based on peer-reviewed literature-derived evidence and international guidelines regarding transmission pathways and reservoir host species [26], [27], [28], [29], [30], [31], [32], [33], [34], [35], [36]:

• High exposure score (3): HAC and reservoir host species strongly associated with pathogen transmission.
• Medium exposure score (2): HAC and reservoir host species with plausible but less consistent evidence.
• Low exposure score (1): HAC and reservoir host species occasionally documented as transmission route.
• No exposure score (0): No evidence of zoonotic transmission.

**Table 1:** Zoonotic exposure index for human-animal contacts.

	3	2	1	0
Rabies	Any direct contact to bats, bites from dogs and wild carnivores	Any direct contact to dogs and wild carnivores	Bites from primates or cats	No direct contact with animals known to transmit rabies
Brucellosis	Raising cattle, sheep, goats and pigs	Raising camelids and dogs; consumption of raw or undercooked meat from cattle, sheep, goats, camelids and pigs, wild deer, chancho monte or carnivores; Handling and slaughtering of cattle, sheep, goats, and pigs; Hunting and slaughtering wild deer, chancho monte or wild carnivores	Handling dogs; handling and slaughtering camelids	No contacts relevant for *Brucella* spp. transmission as described in columns 1–3 (may include contact with pets or animals not involved in handling, raising, hunting, slaughtering, or consumption of raw animal products)
Tuberculosis	Slaughtering cattle; hunting and slaughtering deer; consumption of raw or undercooked meat from cattle and deer	Direct or indirect contact with cattle, goats, camelids, deer and rodents	Direct contact with dogs and cats	No contacts relevant for *Mycobacterium bovis* transmission as described in columns 1–3 (may include e.g. indirect contact with dogs and cats or raw or undercooked consumption of meat from animals other than cattle and deer)

Human–animal contacts were classified according to their documented relevance for transmission of rabies, brucellosis, and bovine tuberculosis. Scores ranged from 0 (no exposure) to 3 (high exposure). Weighting was based on peer-reviewed literature-derived evidence and international guidelines regarding known reservoir hosts and transmission pathways [26], [27], [28], [29], [30], [31], [32], [33], [34], [35], [36].

**Rabies**, one of the deadliest diseases caused by the *Lyssa virus* spp. With 60,000 human deaths yearly, it is primarily transmitted through saliva, most likely through bites from dogs, bats, or wild carnivores [26], [27], and with rare reported cases of transmission from non-human primates and cats [28], [29], [30]. The WHO classifies direct contact with intact skin as no rabies exposure. Direct contact with uncovered skin or minor scratches is classified as exposure, and severe exposure involves transdermal bites or scratches, or any direct contact with bats [31]. Consequently, bites from dogs and wild carnivores and any direct contact with bats were classified as high exposure (3). Direct contact with dogs and wild carnivores without bites was classified as medium exposure (2), reflecting possible saliva exposure through scratches or mucosal contact. In contrast, transmission from cats and non-human primates has been documented only sporadically and is considered comparatively uncommon, usually representing spillover infections rather than sustained reservoir hosts. Therefore, bites from cats and primates were classified as low exposure (1). Contacts without direct interaction with recognized rabies-transmitting species were assigned a score of 0.

**Brucellosis** caused by *Brucella* spp. goes along with reproductive and financial losses in agriculture. Infected humans typically show chronic flu-like symptoms. The major transmission route is through contact with vaginal excretions and aborted material or consumption of raw milk or undercooked meat. Also, other direct contact, such as handling, or indirect contact, such as inhalation of contaminated aerosols, represents a risk of exposure. The disease exemplifies a classic interface between wildlife, domestic animals, and humans, where transmission cycles are influenced by ecological, agricultural, and socioeconomic factors. Domestic species like cattle, sheep, goats, and swine are primary hosts, and their interactions with wildlife—often intensified by habitat encroachment, shared grazing lands, and limited biosecurity—facilitate pathogen transmission. Dogs and camelids have also been shown to transmit the disease to humans, mainly through contact with reproductive discharge when raising [26], [32], [33], [34], [35]. Highest scores (3) were assigned to raising cattle, sheep, goats, and pigs because these species are major reservoirs, and daily husbandry increases repeated exposure to secretions and birth products. Medium scores (2) were assigned to handling, slaughtering, hunting, or consuming raw or undercooked meat from susceptible domestic and wild animals, reflecting substantial but generally less continuous exposure. Dogs and camelids were assigned lower scores because zoonotic transmission is documented but less common. Contacts without documented relevance for *Brucella* spp. transmission received a score of 0.

**Bovine tuberculosis** caused by *Mycobacterium bovis*, affects cattle as the main reservoir and many other domestic and wild species as spillover hosts. Humans are infected mainly by consuming raw milk, raw or undercooked meat, inhaling infectious aerosols, or through skin wounds when handling infected animals. Its multi-host nature and frequent presence at the human–animal interface make control challenging [36]. Highest risk scores (3) were assigned to HAC, which are strongly associated with *M. bovis* infection, such as slaughtering cattle, consuming raw or undercooked meat. Medium risk scores (2) were assigned when infection is regularly documented through direct and indirect contact with cattle, goats, camelids, or wild reservoirs such as deer and rodents, and low risk scores (1) were assigned when occasional spillover was documented, such as direct contact with dogs and cats [36].

For each participant, pathogen-specific exposure scores were calculated by summing all weighted HAC-species combinations reported. Thus, participants reporting multiple high exposure practices accumulated higher scores than those reporting fewer or lower exposure contacts. The resulting indices represent cumulative proxy indicators of theoretical zoonotic exposure opportunity rather than validated measures of infection probability.

### 2.6 Statistical analysis

All analyses were conducted using R and structured in two parts:

**Part 1 – Human-animal contact patterns:** To examine the distribution of HAC, a categorical analysis was conducted. Participants were grouped into four mutually exclusive categories: (1) contact with both domestic and wild animals, (2) contact with domestic animals only, (3) contact with wild animals only, and (4) no contact with animals. Frequencies and proportions were calculated for each category, stratified by country, area, age group, and gender. Patterns of HAC were further visualized by frequencies of mode of contact and animal types using heatmaps.

**Part 2 – Zoonotic exposure:** Firstly, unadjusted mean scores were calculated for rabies, brucellosis, and bovine tuberculosis exposure scores, stratified by sociodemographic and knowledge-related variables. Secondly, multivariable linear regression models were used to assess association between these variables. Model selection was guided by model fit using Akaike Information Criterion. Both mixed-effects models with random intercepts for country and models including country as a fixed effect were evaluated. To assess the robustness of the weighting approach, we conducted a sensitivity analysis using an unweighted version of the index, in which all exposure items were assigned equal weight. Correlation between the weighted and unweighted indices was assessed, and multivariable regression models were repeated using the unweighted index to evaluate whether the main patterns of association were consistent.

## 3 Results

### 3.1 Sociodemographics

A total of 2389 persons participated in the study, thereof 922 in Bolivia, 610 in Chile and 857 in Guatemala. Overall, 1265 participants were female, 1117 male and 6 identified as other. 565 adolescents, 1007 Adults and 811 Elderly. Table 2 shows that 2207 (92.4%) participants reported contact with domestic animals and 753 (31.5%) reported contact with wild animals. Except from 11 participants, all of the persons in contact with wild animals were also in contact with domestic animals (*N* = 742). 171 (7.2%) persons reported no contact with any animal. All participants in Bolivia reported contact with domestic animals, with 31.9% also reporting wild animal contact. In Chile, most participants (74.6%) reported contact with domestic animals only, while the highest proportion of contact with both domestic and wild animals was observed in Guatemala (43.1%). Across **areas**, proportions of contact were relatively evenly distributed. 30–32% of participants in urban, rural, and protected areas reported contact with both domestic and wild animals, while between 58 and 64% reported contact with domestic animals only. Regarding **age groups**, adolescents reported the highest proportion of dual contact with domestic and wild animals (43.9%), while adults and elderly participants more often reported contact with domestic animals only (62.8% and 67.2%, respectively).

**Table 2:** Absolute and relative frequencies of sociodemographic characteristics of the study population by contact with domestic and wild animals.

*N* = 2389	Missing	Contact with domestic and wild animals *N* = 742		Contact with domestic animals only *N* = 1465		Contact with wild animals only *N* = 11		No contact with animals *N* = 171	
	N	n	%	n	%	n	%	n	%
**Country**	0								
Bolivia		294	31.89	628	68.11	0	0	0	0
Chile		79	12.95	455	74.59	2	0.33	74	12.13
Guatemala		369	43.06	382	44.57	9	1.05	97	11.32

**Area**	0								
urban		284	31.52	523	58.05	5	0.55	89	9.88
rural		266	30.12	555	62.85	1	0.11	61	6.91
Protected area		192	31.74	387	63.97	5	0.83	21	3.47

**Age group**^a^	6								
Asolescents		248	43.89	284	50.27	5	0.88	28	4.96
Adults		291	28.9	632	62.76	5	0.5	79	7.85
Elderly		202	24.91	545	67.2	1	0.12	63	7.77

**Gender**	1								
Female		303	23.95	878	69.41	2	0.16	82	6.48
Male		436	39.03	586	52.46	9	0.81	86	7.7
Other		3	50	1	16.67	0	0	2	33.33

a Age groups were defined as follows: Adolescents (10-17 years), Adults (18-60 years), and Elderly (61 years and older).

**Gender** differences were also evident: males were more likely to report contact with both domestic and wild animals (39.0%), whereas females more frequently reported contact with domestic animals only (69.4%).

### 3.2 Patterns of domestic animal contacts

**Fig. 2:**
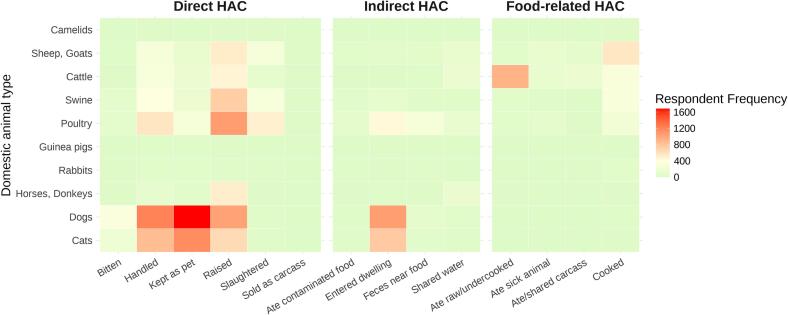
Respondent frequency of domestic animal contacts by animal type. The heatmap visualizes the frequency of reported human-animal contacts (HAC) over the preceding 12 months by animal type, categorized into three potential zoonotic transmission pathways: direct, indirect, and food-borne. The x-axis represents different modes of contact, while the y-axis lists the domestic animal types. Greater color intensity, with red representing the highest values, indicates more frequently reported HAC and thus higher levels of potential zoonotic exposure. These values reflect self-reported exposure opportunities rather than direct measures of infection risk. (For interpretation of the references to color in this figure legend, the reader is referred to the web version of this article.)

Fig. 2 demonstrates, that overall, the highest frequencies of interactions - as reported by participants over the preceding 12-month period - with domestic animals were of **direct** nature, with having animals as pets, handling, and raising animals being common practices. Dogs and cats showed the highest frequencies of direct contact, with many respondents reporting having them as pets (dogs *N* = 1681; cats *N* = 1150). Livestock, such as poultry, swine, sheep and goats, and cattle, were primarily associated with raising (poultry *N* = 1054; swine *N* = 735; sheep and goats *N* = 522; cattle *N* = 469) and handling (poultry *N* = 572; swine *N* = 370; sheep and goats *N* = 276; cattle *N* = 305). Slaughtering practices were most frequently reported for poultry (*N* = 492), swine (*N* = 299), sheep and goats (*N* = 290). Bites from animals were also documented, predominantly from dogs (*N* = 342). **Indirect** contacts included interactions such as animals entering or living inside of the dwellings and having seen feces near the food. Dogs and cats, as well as poultry showed notable frequencies of entering the dwelling (dogs *N* = 1048; cats *N* = 760; poultry *N* = 439). Feces near food was mainly regarding poultry (*N* = 280). Consumption of food touched or damaged by animals was rarely reported. Water sources were shared with cattle (*N* = 144), horses and donkeys (*N* = 137), poultry (*N* = 127), sheep and goats (*N* = 121).

**The food-related** contacts showed lower overall frequencies compared to direct and indirect contact, but it remained relevant for specific animal types. Consumption of undercooked or raw meat was reported for cattle (*N* = 913). Consumption of cooked animals was reported for all livestock: sheep and goats *N* = 563, cattle (*N* = 311), swine (*N* = 296) and poultry (*N* = 235).

### 3.3 Patterns of wild animal contacts

**Fig. 3:**
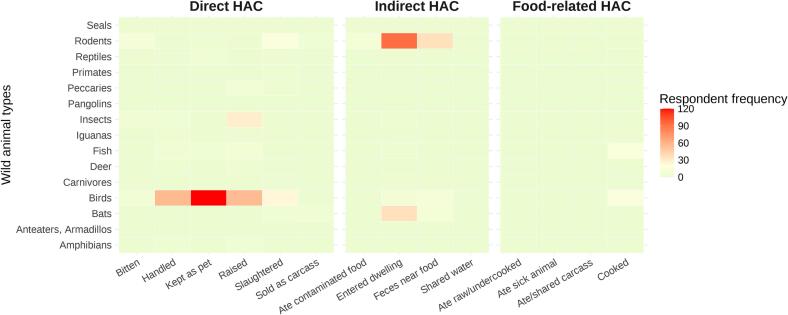
Respondent frequency of wild animal contacts by animal types. The heatmap visualizes the frequency of reported human-animal contacts (HAC) over the preceding 12 months by animal types, categorized into three potential zoonotic transmission pathways: direct, indirect, and food-borne. The x-axis represents different modes of contact, while the y-axis lists the wild animal types. Greater color intensity, with red representing the highest values, indicates more frequently reported HAC and thus higher levels of potential zoonotic exposure. These values reflect self-reported exposure opportunities rather than direct measures of infection risk. (For interpretation of the references to color in this figure legend, the reader is referred to the web version of this article.)

Compared to domestic animals, the overall contact with wild animals – as reported by participants over the preceding 12-month period - were considerably lower (Fig. 3). Wild birds were the most common animal type **directly** in contact with study participants through handling (*N* = 56), keeping them as pets (*N* = 120) and raising (*N* = 55). Furthermore, birds (*N* = 24) and rodents (*N* = 16) were slaughtered, and insects (*N* = 29) raised. **Indirect** interactions included rodents (*N* = 93) and bats (*N* = 36) entering the dwellings, fecal contamination near food from rodents (N = 36). **Food-related** contact with wild animals were rarely reported. The most notable findings include limited reports of cooking birds (N=) and fish (*N* = 14). All other reported contact with wild animals were below *N* = 10.

Patterns of contact with both domestic and wild animals are provided in the Supplementary Material (Fig. S2).

### 3.4 Zoonotic exposure: rabies

The mean rabies exposure score varied across countries and participant characteristics (Fig. S3). Participants in Bolivia showed the highest mean exposure score, followed by those in Guatemala and Chile. Participants who had received zoonosis training had lower exposure scores than those who had not. Adolescents showed higher exposure scores compared with adults and elderly participants. Participants with tertiary education had lower scores than those with no, primary, or secondary education. Differences across areas of residence, monthly income, and gender were small.

**Fig. 4:**
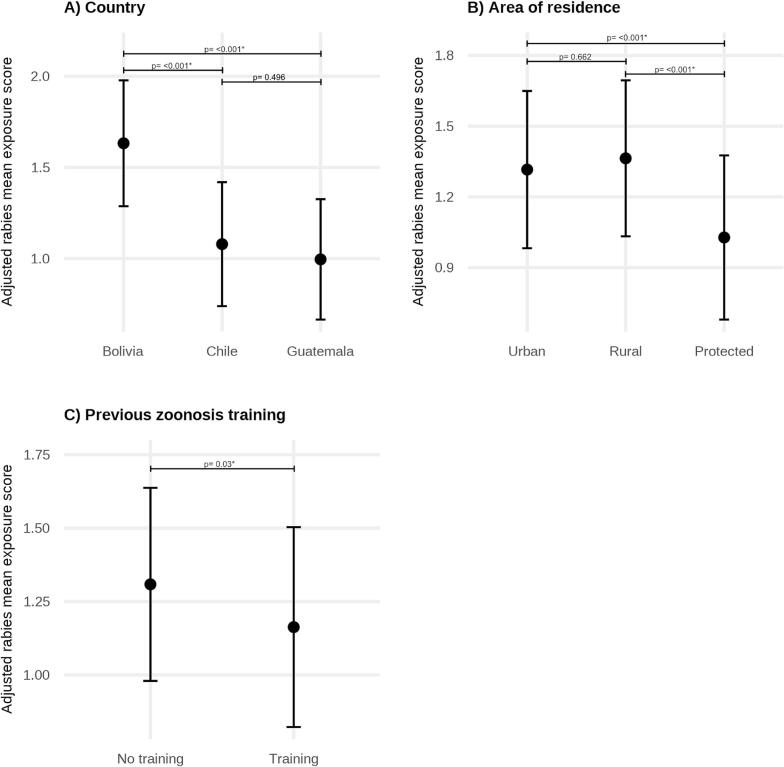
Adjusted mean rabies exposure scores by (A) country, (B) area of residence, and (C) previous zoonosis training. Estimates are derived from a multivariable linear regression model adjusting for age group, gender, education, monthly income, and the remaining variables shown. Points represent adjusted mean exposure scores and error bars indicate 95% confidence intervals. Model estimates are presented in Table S4.

Multivariable linear regression models that included all predictors demonstrated substantially better fit than those that did not. Models including country and area of residence as fixed effects showed better fit than mixed-effects models with random intercepts and were therefore retained for the final analysis. The final adjusted model demonstrated that country, previous zoonosis training, and area of residence were significantly associated with rabies exposure scores (Fig. 4, Table S4). Compared to Bolivia, exposure scores were significantly lower in Chile (β = −0.55, 95% CI: −0.73 to −0.38, *p*-value: <0.001) and Guatemala (β = −0.64, 95% CI: −0.80 to −0.47, p-value: <0.001). Participants who had received zoonosis training had lower adjusted exposure scores compared to those without training (β = −0.15, 95% CI: −0.28 to −0.01, p-value: 0.03). No significant difference was observed between rural and urban settings. However, participants living in protected areas had significantly lower exposure scores compared to both urban (β = −0.29, 95% CI: −0.47 to −0.11, p-value: <0.001) and rural areas (β = −0.34, 95% CI: −0.52 to −0.15, p-value: <0.001). No significant associations were observed for age group, gender, education, or monthly income after adjustment for other variables.

### 3.5 Zoonotic exposure: brucellosis

In the unadjusted analysis (Fig. S5), adolescents showed the highest mean exposure score. Participants living in protected areas reported higher scores than those in urban or rural settings. Education level and monthly income were inversely associated with exposure: lower-income groups and participants with lower education levels reported higher exposure than middle- and higher-income groups and higher-educated participants. Furthermore, prior training on zoonoses was linked to lower exposure scores. Gender differences were small.

**Fig. 5:**
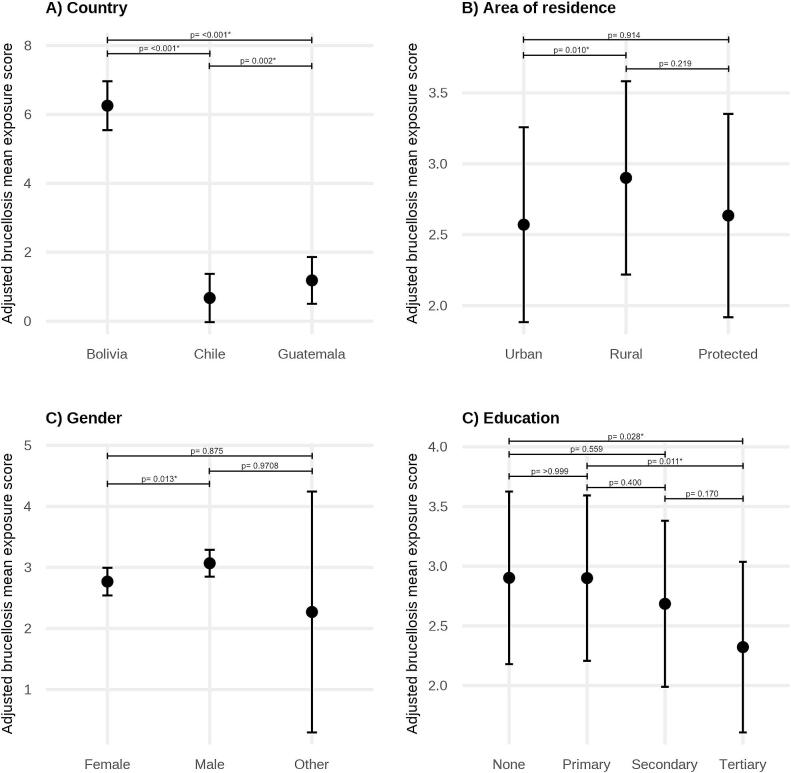
Adjusted mean brucellosis exposure scores by (A) country, (B) area of residence, (C) gender, and D) education. Estimates are derived from a multivariable linear regression model adjusting for age group, previous zoonosis training, monthly income, and the remaining variables shown. Points represent adjusted mean exposure scores and error bars indicate 95% confidence intervals. Model estimates are presented in Table S6.

As for rabies, multivariable models for brucellosis including country as a fixed effect showed better fit than mixed-effects models and were therefore retained for the final analysis. In the adjusted model, country, gender, education, and area of residence were significantly associated with brucellosis exposure scores (Fig. 5, Table S6). Compared to Bolivia, adjusted exposure scores were substantially lower in Chile (β = −5.6, 95% CI: −5.9 to −5.2, *p*-value: <0.001) and Guatemala (β = −5.1, 95% CI: −5.4 to −4.7, p-value: <0.001). Male participants had higher exposure scores compared to female participants (β = 0.30, 95% CI: 0.05 to 0.55, p-value: 0.013). With respect to education, tertiary education was associated with lower exposure scores than no education (β = −0.58, 95% CI: −1.1 to −0.04, p-value: 0.028) and primary education (β = −0.58, 95% CI: −1.1 to −0.10, p-value: 0.011). Regarding area of residence, living in rural areas was associated with higher exposure scores than in urban settings (β = 0.33, 95% CI: 0.06 to 0.60, p-value: 0.010). No significant associations were observed for age group, monthly income, or previous zoonosis training after adjustment for other variables.

### 3.6 Zoonotic exposure: Bovine tuberculosis

In the unadjusted analysis (Supplementary Fig. S7), adolescents showed the highest exposure scores compared with adults and the elderly. Female participants were slightly more exposed than male participants. Participants living in protected settings reported markedly higher scores than those in urban or rural settings. Participants with tertiary education reported lower scores than those with lower or no education. Low-income participants showed higher exposure scores than higher-income participants. Finally, those previously trained on zoonoses showed reduced exposure scores.

**Fig. 6:**
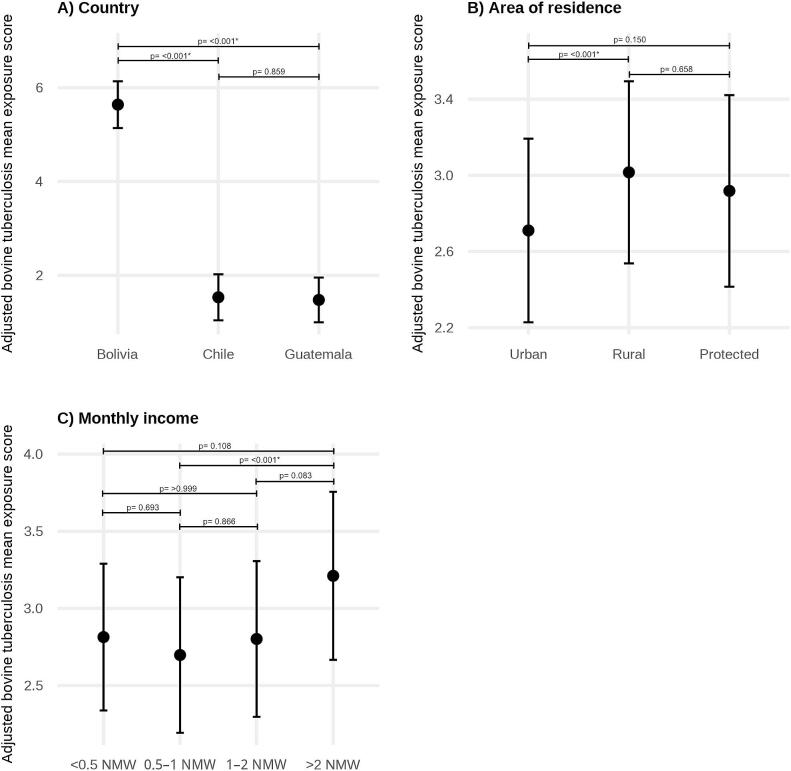
Adjusted mean bovine tuberculosis exposure scores by (A) country, (B) area of residence, and (C) monthly income. Estimates are derived from a multivariable linear regression model adjusting for age group, gender, previous zoonosis training, education, and the remaining variables shown. Points represent adjusted mean exposure scores and error bars indicate 95% confidence intervals. Model estimates are presented in Table S8. NMW: National minimum wage.

As in previous models, models including country as a fixed effect showed better fit than mixed-effects models and were therefore retained for the final analysis. In the adjusted model, country, monthly income, and area of residence were significantly associated with tuberculosis exposure scores (Fig. 6, Table S8). Compared to Bolivia, estimated exposure scores were significantly lower in Chile (β = −4.1, 95% CI: −4.4 to −3.9, *p*-value: <0.001) and Guatemala (β = −4.2, 95% CI: −4.4 to −3.9, p-value: <0.001). Regarding income, findings showed that the highest income category was associated with higher exposure scores than the lowest income category (β = 0.51, 95% CI: 0.04 to 0.98, p-value: 0.026). Regarding area of residence, participants living in rural areas had higher estimated exposure scores than those in urban areas (β = 0.31, 95% CI: 0.12 to 0.49, p-value: <0.001). No significant associations were observed for age group, gender, education, or previous zoonosis training after adjustment for other variables.

The weighted and unweighted exposure indices were highly correlated across all pathogens (Spearman's ρ = 1.00 for rabies and brucellosis, and ρ = 0.95 for bovine tuberculosis; all *p* < 0.001). Analyses using the unweighted index yielded similar patterns across sociodemographic groups, and multivariable models showed comparable directions and magnitudes of associations.

## 4 Discussion

HAC is a critical driver of zoonotic diseases, yet empirical, population-based evidence remains limited, particularly from Latin America. This study contributes new insights by systematically characterizing HAC patterns and estimating zoonotic exposure in diverse, wildlife-rich settings across Bolivia, Chile, and Guatemala.

The vast majority of participants reported animal contact, confirming that HAC is not restricted to a small subgroup but is widespread across age and gender. Domestic animal such as dogs, cats, poultry, and swine were the most frequently encountered animal types. The predominance of domestic animal contact compared to wildlife contact in the study is consistent with findings from other studies [13], [37] and these species are known for carrying a wide range of especially endemic zoonotic pathogens [3], [4], [5]. One-third reported HAC with wild animals, predominantly wild birds, rodents, and bats. Almost all individuals with wildlife HAC also reported contact with domestic animals, highlighting the interconnectedness of wildlife–livestock–human interfaces and the potential for cross-species transmission of multi-host pathogens such as rabies, brucellosis, and bovine tuberculosis.

At first sight, HAC with ‘wild’ birds ‘kept as pets’ may appear contradictory. However, additional qualitative insights from interviews conducted within the same study suggest that species such as chachalacas, parrots, and turkeys in Guatemala are originally from the wild but are frequently captured and later kept for food or cultural purposes (unpublished qualitative results).

Direct contacts, such as pet ownership, handling, and slaughtering, dominated the reported interactions, while indirect HAC occurred less frequently. Notably, the consumption of undercooked or raw beef was common, a known transmission route for zoonoses such as *Taenia saginata*, *Salmonella* spp., *Mycobacterium bovis*, and *Brucella* spp., which are all relevant in Latin America.

Across multivariable models, country and area of residence consistently emerged as key factors shaping estimated exposure patterns, underscoring the importance of local (agro-)ecological and socio-cultural contexts at the human–animal interface. In contrast, individual-level sociodemographic characteristics such as age, gender, education, and income as well as prior knowledge on zoonoses showed less consistent associations after adjustment, suggesting that zoonotic exposure is primarily structured by contextual rather than individual determinants. This contrasts with findings from other studies, which reported that sociodemographic factors such as gender, age, and education were associated with more risk-averse attitudes and preventive behaviors [15], [38]. In our study, adolescents showed the highest mean exposure across all zoonoses, however this association was no longer significant after multivariable adjustment. This may reflect greater involvement in animal care and reduced risk perception and knowledge, as shown in prior studies [39]. The attenuation of the association after adjustment suggests that these differences are likely driven by contextual factors, such as livelihood practices and environmental conditions, rather than by age itself.

Exposure patterns differed between pathogens, which may reflect their distinct transmission pathways and epidemiological profiles. For rabies, previous zoonosis training was associated with lower exposure scores, suggesting that behavioral awareness and risk perception may influence high-risk contacts, such as animal bites. In contrast, brucellosis exposure was associated with male gender and lower education. However, gender distribution was overall balanced, suggesting that zoonotic risks are shared across groups. For bovine tuberculosis, higher exposure in rural areas and among higher-income groups may reflect patterns of livestock ownership, slaughtering practices, and consumption behaviors. Notably, Bolivia has been identified as a country with comparatively limited research and surveillance capacity for zoonotic diseases [16]. In this context, the highest exposure levels observed in Bolivia, even after adjustment for area of residence and sociodemographic variables, underscore the importance of strengthening Bolivian research on zoonotic diseases. Importantly, the results should be interpreted as reflecting patterns of potential zoonotic exposure, based on self-reported contact behaviors, rather than direct measures of infection risk.

This study fills an important knowledge gap by systematically describing HAC patterns in biodiverse Latin American settings. Strengths include its multi-country design, covering diverse ecological and cultural contexts, and the use of standardized, piloted survey tools. The zoonotic exposure index integrates evidence-based weighting of high-risk behaviors from peer-reviewed literature across multiple regions, providing a transparent framework for exposure estimation.

However, several limitations should be noted. First, the questionnaire covered only a limited set of exposure pathways and did not include the consumption of unpasteurized milk. As a result, important exposure routes for zoonotic pathogens such as *Brucella* spp. and *Mycobacterium bovis* could not be assessed, and the zoonotic exposure index may therefore underestimate exposure for some participants. This omission reflects the questionnaire's original focus on wildlife-related practices, in which milk consumption was not considered a primary exposure pathway.

Second, although participants reported whether specific HAC had occurred during the preceding 12 months, the questionnaire did not capture the frequency or intensity of these contacts. We were therefore unable to distinguish between occasional and repeated exposures, which may differ substantially in their relevance for zoonotic transmission. Other studies usually assumed daily interaction for livestock owners due to daily care of the animals [40], [41].

Furthermore, certain regions and populations were underrepresented in this study. For instance, no communities located within protected areas were included in Chile. In addition, the exclusion of children in Chile—due to lack of parental consent—may have biased age-related findings. The study relied on self-reported contacts over a 12-month period, which is subject to recall bias, although they are widely used in risk factor studies [13]. Additionally, reporting bias cannot be excluded, as socially sensitive behaviors (e.g., hunting or wildlife handling) may have been underreported.

The study design was cross-sectional, based exclusively on questionnaires with neither laboratory nor microbiological sampling nor medical diagnoses. Thus, pathogen circulation in local animal populations or medical conditions of the participants could not be assessed. As highlighted elsewhere, zoonotic risk assessment remains challenging even with pathogen surveillance data [42]. Other studies have demonstrated association between HAC and symptoms [13]. Consequently, our findings should be interpreted as a baseline description of contact patterns and theoretical exposure potential, not evidence of causality. The weighting scheme of the zoonotic exposure score was informed by previous studies. While these studies only partly derive from the specific cultural and ecological context of the study population, they provide a biologically plausible and literature-based framework for differentiating exposure levels. While a simpler unweighted index assumes equal relevance of all contact types, this does not reflect established transmission pathways for the selected pathogens. In sensitivity analyses using an unweighted index, we observed similar overall patterns, suggesting that the findings are robust and not driven by the specific weighting scheme. At the same time, the weighted index provides a more conceptually grounded representation of potential exposure by differentiating between contact types with varying transmission relevance.

Despite an extensive search, no directly comparable studies could be identified for the specific regions across Bolivia, Chile, and Guatemala investigated. Therefore, the findings presented here contribute novel insights regarding zoonotic risk for these geographic contexts. However, zoonotic exposure represents only one component of overall zoonotic risk. The magnitude of this risk is highly context-dependent, as it reflects the presence and circulation of specific pathogens in a given area — also referred to as zoonotic hazard — as well as the level of host vulnerability [22], [43]. For instance, direct contact with dogs may pose minimal risk for rabies transmission in areas where canine rabies is not endemic and vaccination coverage among both humans and animals is high. In contrast, such contact represents a high risk in regions where rabies is endemic, with unvaccinated dogs and the lack of timely (post-exposure) prophylaxis for humans, which increases vulnerability in both. Moreover, vulnerability itself influences risk outcomes even under similar exposure levels, as host-related factors—such as prior immunity, age, pregnancy, and comorbidities—modulate both the likelihood of infection and the severity of disease. Therefore, the index should be interpreted as a proxy indicator of theoretical exposure, based on the type and frequency of HAC, rather than as a validated measure of zoonotic infection risk.

Finally, participants were nested within countries and communities, and observations may therefore not be fully independent. Although models including country and area of residence as fixed effects showed better fit, residual clustering at the community level cannot be excluded. This may have led to underestimation of variability, and results should be interpreted with this limitation in mind.

Despite these limitations, the study offers important insights for One Health interventions. Our findings highlight that human–animal contact occurs at overlapping interfaces between wildlife and domestic animals, rather than within separate domains. This suggests that the common narrative of zoonotic diseases as primarily a wildlife-related problem should be broadened to explicitly include the interconnected roles of domestic animals. Accordingly, interventions may not focus solely on wildlife but instead address the full human–animal–environment interface. Direct interactions, including ownership, handling, and slaughtering, were the most common, highlighting the need for targeted preventive measures during routine animal handling. Our results further indicate that context-specific factors, such as country and area of residence, are more important than individual factors. Although age, gender, income, and education were not consistently independently associated with all of the exposure scores after adjustment, adolescents and individuals with lower income or education may still represent important target groups for interventions, as they are more frequently involved in animal-related practices, as demonstrated in descriptive analysis. Moreover, the consistently higher exposure levels observed in Bolivia emphasize the need for increased research and surveillance efforts in underrepresented settings. For further research, we suggest applying an indicator framework—pathogen circulation and contact intensity—that prioritizes interventions at the geographic, social, and cultural levels. Ultimately, predictive models that combine human behavioral data (such as from this study) with pathogen surveillance, ecological dynamics, environmental factors, and host vulnerability will be critical for identifying high-risk interfaces and guiding prevention efforts at the human-animal-environment interface.

## Declaration of generative AI and AI-assisted technologies in the manuscript preparation process

During the preparation of this work, the authors used ChatGPT (OpenAI; GPT-5.1) and DeepL to support language refinement. After using these tools, the authors reviewed, edited, and validated all content as needed and take full responsibility for the accuracy, integrity, and originality of the published article.

## CRediT authorship contribution statement

**Caroline Kuhn:** Writing – original draft, Visualization, Software, Project administration, Methodology, Formal analysis, Data curation, Conceptualization. **Katja Radon:** Writing – review & editing, Validation, Project administration, Funding acquisition, Data curation, Conceptualization. **Fabiana Marcela Pérez Morales:** Writing – review & editing, Investigation. **Marcia Adler:** Writing – review & editing, Investigation. **Carlos Fernando Pinto Navia:** Writing – review & editing, Investigation. **María Soledad Burrone:** Writing – review & editing, Validation. **Denise Siqueira de Carvalho:** Writing – review & editing, Investigation, Data curation. **María Teresa Solis Soto:** Writing – review & editing, Validation, Supervision, Project administration, Funding acquisition, Data curation.

## Ethical statement

Ethical approval was obtained from the Ethics Committees at the Ludwigs-Maximilians-University in Munich, Germany (ID: 22–0254); the Universidad Mayor de Simon in Cochabamba, Bolivia (ID: CEI-FM-UMSS); the Universidad O'Higgins, Rancagua, Chile (ID: 014–2022); and the Comite Independiente de Etica K'awil, Guatemala (ID: K'awil - 003-2022). Ethical approval was obtained in all participating countries.

## Funding

This article is part of the study “Knowledge, attitudes, and practices towards the risk of zoonotic diseases, wildlife trade and wildlife consumption in Latin America” and financially supported by a fund of the German Federal Ministry for Economic Collaboration and Development (BMZ) through the International Alliance against Health Risks in Wildlife Trade and coordinated by the GIZ (Gesellschaft für 10.13039/100011259Internationale Zusammenarbeit). Additionally, we receive support from the OH-TARGET (One Health Training and Research Global Network) project, which is part of the Exceed program—Higher Education Excellence in Development Cooperation —funded by the German 10.13039/501100006456Federal Ministry for Economic Cooperation and Development (BMZ) and the 10.13039/501100001655German Academic Exchange Service (DAAD).

## Declaration of competing interest

The authors declare that they have no known competing financial interests or personal relationships that could have appeared to influence the work reported in this paper.

## Data Availability

Data will be made available on request.

## References

[1] Daszak P., Cunningham A.A., Hyatt A.D. (2001). Anthropogenic environmental change and the emergence of infectious diseases in wildlife. Acta Trop..

[2] Jones K.E., Patel N.G., Levy M.A. (2008). Global trends in emerging infectious diseases. Nature.

[3] Otte J., Pica-Ciamarra U. (2021). Emerging infectious zoonotic diseases: the neglected role of food animals. One Health.

[4] Klous G., Huss A., Heederik D.J.J. (2016). Human–livestock contacts and their relationship to transmission of zoonotic pathogens, a systematic review of literature. One Health.

[5] Magouras I., Brookes V.J., Jori F. (2020). Emerging zoonotic diseases: should we rethink the animal–human interface?. Front. Vet. Sci..

[6] Caserta L.C., Frye E.A., Butt S.L. (2024). Spillover of highly pathogenic avian influenza H5N1 virus to dairy cattle. Nature.

[7] Stevenson M., Halpin K. (2021). Emerging and endemic zoonotic diseases: surveillance and diagnostics. Rev. Sci. Tech..

[8] Grace D., Mutua F., Ochungo P. (2012). https://hdl.handle.net/10568/21161.

[9] Maxwell M.J., Freire De Carvalho M.H., Hoet A.E. (2017). Building the road to a regional zoonoses strategy: a survey of zoonoses programmes in the Americas. PLoS One.

[10] Fournié G., Kearsley-Fleet L., Otte J. (2015). Spatiotemporal trends in the discovery of new swine infectious agents. Vet. Res..

[11] Elsohaby I., Villa L. (2023). Zoonotic diseases: understanding the risks and mitigating the threats. BMC Vet. Res..

[12] Ahmed A.N., Fornace K.M., Iwamura T. (2025). Human animal contact, land use change and zoonotic disease risk: a protocol for systematic review. Syst. Rev..

[13] Yadana S., Cheun-Arom T., Li H. (2022). Behavioral–biological surveillance of emerging infectious diseases among a dynamic cohort in Thailand. BMC Infect. Dis..

[14] Bhatia B., Sonar S., Khan S. (2024). Pandemic-proofing: intercepting zoonotic spillover events. Pathogens.

[15] Palomares Velosa J.E., Riaño Sánchez S., Martínez Marín A. (2022). Prevention of exposure to zoonoses in rural Latin America: social ecological factors in a diverse regional context. One Health.

[16] Reyes R., Yohannessen K., Ayala S. (2019). Estimaciones de la distribución espacial del riesgo relativo de mortalidad por las principales zoonosis en Chile: enfermedad de Chagas, hidatidosis, síndrome cardiopulmonar por hantavirus y leptospirosis. Rev. Chilena Infectol..

[17] Sharmin S., Raj A., Zawad M.A. (2026). Chapare virus: a re-emerging zoonotic arenavirus with limited preparedness. Rev. Med. Virol..

[18] Taylor E., Aguilar-Ancori E.G., Banyard A.C. (2023). The Amazonian tropical bites research initiative, a hope for resolving zoonotic neglected tropical diseases in the one health era. Int. Health.

[19] De Macedo Couto R., Santana G.O., Ranzani O.T. (2022). One health and surveillance of zoonotic tuberculosis in selected low-income, middle-income and high-income countries: a systematic review. PLoS Negl. Trop. Dis..

[20] Grajeda L.M., McCracken J.P., Berger-González M. (2021). Sensitivity and representativeness of one-health surveillance for diseases of zoonotic potential at health facilities relative to household visits in rural Guatemala. One Health.

[21] Sun Z.-S., Wan E.-Y., Agbana Y.L. (2024). Global one health index for zoonoses: a performance assessment in 160 countries and territories. iScience.

[22] Bernstein A.S., Ando A.W., Loch-Temzelides T. (2022). The costs and benefits of primary prevention of zoonotic pandemics. Sci. Adv..

[23] World Health Organization PREZODE Working-Group. Indicators for the human exposures to zoonotic pathogens. https://cdn.who.int/media/docs/default-source/epi-win/who-prezode-report_epiwin.pptx.pdf?sfvrsn=4fa681f5_1, 2024, (accessed 3. April 2026).

[24] Saylors K.E., Mouiche M.M., Lucas A. (2021). Market characteristics and zoonotic disease risk perception in Cameroon bushmeat markets. Soc. Sci. Med. (1982).

[25] Li H., Daszak F., Chmura A. (2021). Knowledge, attitude, and practice regarding zoonotic risk in wildlife trade, southern China. EcoHealth.

[26] World Organisation for Animal Health, Terrestrial Animal Health Code: Recommendations Applicable to WOAH Listed Diseases, 2023, https://www.woah.org/en/what-we-do/standards/codes-and-manuals/terrestrial-code-online-access/ (accessed 3. April 2026).

[27] Singh R., Singh K.P., Cherian S. (2017). Rabies - epidemiology, pathogenesis, public health concerns and advances in diagnosis and control: a comprehensive review. Vet. Q..

[28] de Lima J.S., Mori E., Kmetiuk L.B. (2023). Cat rabies in Brazil: a growing one health concern. Front. Public Health.

[29] Meske M., Fanelli A., Rocha Felipe (2021). Evolution of rabies in South America and inter-species dynamics (2009–2018). Trop. Med. Infect. Dis..

[30] Devaux C.A., Mediannikov O., Medkour H. (2019). Infectious disease risk across the growing human-non human primate interface: a review of the evidence. Front. Public Health.

[31] World Health Organization (2024). Rabies. https://www.who.int/news-room/fact-sheets/detail/rabies.

[32] Godfroid J. (2017). Brucellosis in livestock and wildlife: zoonotic diseases without pandemic potential in need of innovative one health approaches. Arch. Public Health.

[33] Bonilla-Aldana D.K., Trejos-Mendoza A.E., Pérez-Vargas S. (2023). A systematic review and meta-analysis of bovine brucellosis seroprevalence in Latin America and the Caribbean. New Microbes New Infect..

[34] Wernery U. (2014). Camelid brucellosis: a review. Sci. Tech. Rev..

[35] Weese J.S., Weese H.E. (2025). Brucellosis in humans caused by *Brucella canis*: a scoping review. Can. Vet. J..

[36] Devi K.R., Lee L.J., Yan L.T. (2021). Occupational exposure and challenges in tackling M. Bovis at human–animal interface: a narrative review. Int. Arch. Occup. Environ. Health.

[37] Yadana S., Valitutto M.T., Aung O. (2023). Assessing behavioral risk factors driving zoonotic spillover among high-risk populations in Myanmar. Ecohealth.

[38] Vlaanderen F., Mughini-Gras L., Bourgonje C. (2024). Attitudes towards zoonotic disease risk vary across sociodemographic, communication and health-related factors: a general population survey on literacy about zoonoses in the Netherlands. One Health..

[39] Zucca P., Rossmann M.-C., Dodic M. (2021). What do adolescents know about one-health and zoonotic risks? A school-based survey in Italy, Austria, Germany, Slovenia, Mauritius, and Japan. Front. Public Health.

[40] Burke R.L., Kronmann K.C., Daniels C.C. (2012). A review of zoonotic disease surveillance supported by the armed forces health surveillance center. Zoonoses Public Health.

[41] Klim H., William T., Chua T.H. (2023). Quantifying human-animal contact rates in Malaysian Borneo: influence of agricultural landscapes on contact with potential zoonotic disease reservoirs. Front. Epidemiol..

[42] Wille M., Geoghegan J.L., Holmes E.C. (2021). How accurately can we assess zoonotic risk?. PLoS Biol..

[43] Gibb R., Franklinos L.H.V., Redding D.W. (2020). Ecosystem perspectives are needed to manage zoonotic risks in a changing climate. BMJ.

