# Supplementary material for: Human-animal contact and zoonotic exposure from wild and domestic animals: A cross-sectional study in wildlife-rich areas of Bolivia, Chile, and Guatemala
Source: One Health. 2026 May 8;22:101438. doi: 10.1016/j.onehlt.2026.101438 (PMC13191649; doi:10.1016/j.onehlt.2026.101438)
Supplement: Supplementary material [file mmc1.docx]

# Supplementary Data

Supplementary Material containing Figure S2, Figure S3, Table S4, Figure S5, Table S6, Figure S7, and Table S8


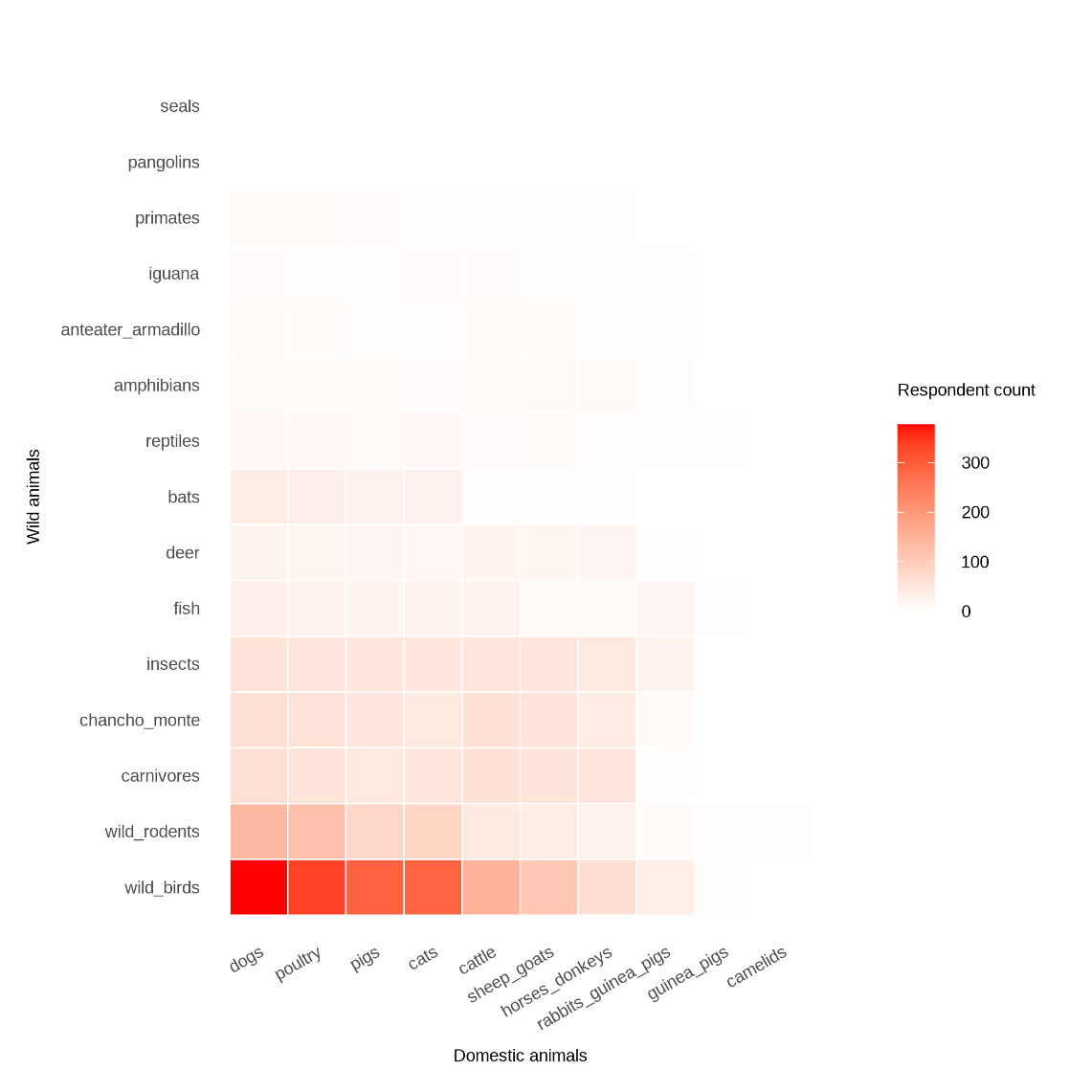


**Figure S2. Heatmap of overlapping human contacts with wild and domestic animals among study participants (N=742).** Each cell represents the number of individuals who reported contact with both a wild and a domestic animal type within the past 12 months, with darker red shades indicating higher counts. Importantly, the figure depicts co-occurrence of participant-reported contacts, not direct interactions between wild and domestic animals, and counts are therefore not directly comparable to the frequencies of wild animal contacts shown in Figure 2.

Among participants reporting contact with both domestic and wild animals within the last 12 months (N = 742), wild birds were most frequently reported in combination with domestic species. The most common overlaps were with dogs (N = 377), poultry (N = 344), pigs (N = 292), cats (N = 288), and cattle (N = 149) (Figure 3). Wild rodents contact were also reported in combination with dog contacts (N = 139) and poultry contacts (N = 124).


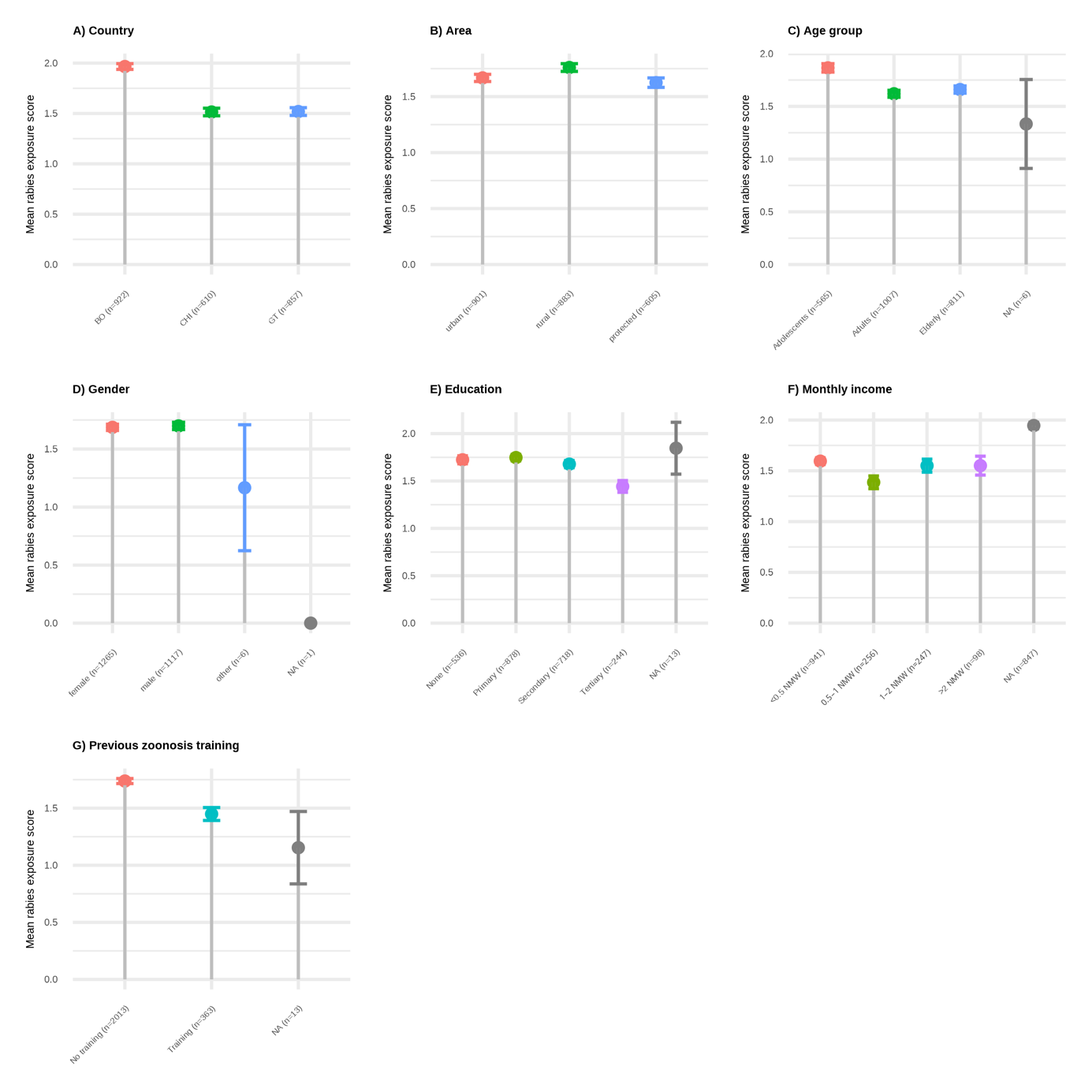


**Figure S3.** Mean rabies exposure scores across sociodemographic and knowledge-related factors for categories of (A) country, (B) area of residence, (C) age group, (D) gender, (E) education, (F) monthly income measured in National Minimum Wage (NMW), and (G) previous zoonoses training. Sample sizes per group are indicated on the x-axis.

**Table S4.** Association between sociodemographic and knowledge-related variables and rabies mean exposure score in multivariable linear regression.

| **Variables** | **Model Estimates (Beta)** | **95% CI^a^** | **p-Value^b^** |
| --- | --- | --- | --- |
| Country |  |  |  |
| Chile - Bolivia | -0.55 | -0.73, -0.38 | **<0.001** |
| Guatemala - Bolivia | -0.64 | -0.80, -0.47 | **<0.001** |
| Guatemala - Chile | -0.08 | -0.26, 0.09 | 0.496 |
| Age group |  |  |  |
| Adults - Adolescents | -0.11 | -0.33, 0.11 | 0.451 |
| Elderly - Adolescents | -0.09 | -0.32, 0.14 | 0.639 |
| Elderly - Adults | 0.02 | -0.12, 0.17 | 0.920 |
| Gender |  |  |  |
| male - female | 0.03 | -0.09, 0.15 | 0.801 |
| other - female | -0.87 | -2.0, 0.28 | 0.179 |
| other - male | -0.90 | -2.1, 0.25 | 0.156 |
| Education |  |  |  |
| Primary - None | 0.11 | -0.07, 0.30 | 0.405 |
| Secondary - None | 0.09 | -0.12, 0.29 | 0.704 |
| Secondary - Primary | -0.03 | -0.20, 0.15 | 0.978 |
| Tertiary - None | 0.03 | -0.24, 0.29 | 0.994 |
| Tertiary - Primary | -0.09 | -0.32, 0.15 | 0.767 |
| Tertiary - Secondary | -0.06 | -0.28, 0.16 | 0.892 |
| Monthly income^c^ |  |  |  |
| 0.5–1 NMW - <0.5 NMW | -0.11 | -0.30, 0.08 | 0.456 |
| 1–2 NMW - <0.5 NMW | 0.01 | -0.20, 0.23 | 0.999 |
| 1–2 NMW - 0.5–1 NMW | 0.12 | -0.12, 0.36 | 0.560 |
| >2 NMW - <0.5 NMW | 0.08 | -0.23, 0.39 | 0.907 |
| >2 NMW - 0.5–1 NMW | 0.19 | -0.14, 0.52 | 0.437 |
| >2 NMW - 1–2 NMW | 0.07 | -0.24, 0.38 | 0.939 |
| Previous training |  |  |  |
| Training - No training | -0.15 | -0.28, -0.01 | **0.030** |
| Area of Residence |  |  |  |
| rural - urban | 0.05 | -0.08, 0.18 | 0.662 |
| protected - urban | -0.29 | -0.47, -0.11 | **<0.001** |
| protected - rural | -0.34 | -0.52, -0.15 | **<0.001** |
| ^a^Confidence Interval; ^b^Significant effects are marked in bold (threshold : 0.05) ; ^c^Monthly income was categorized relative to the national minimum wage. | | | |


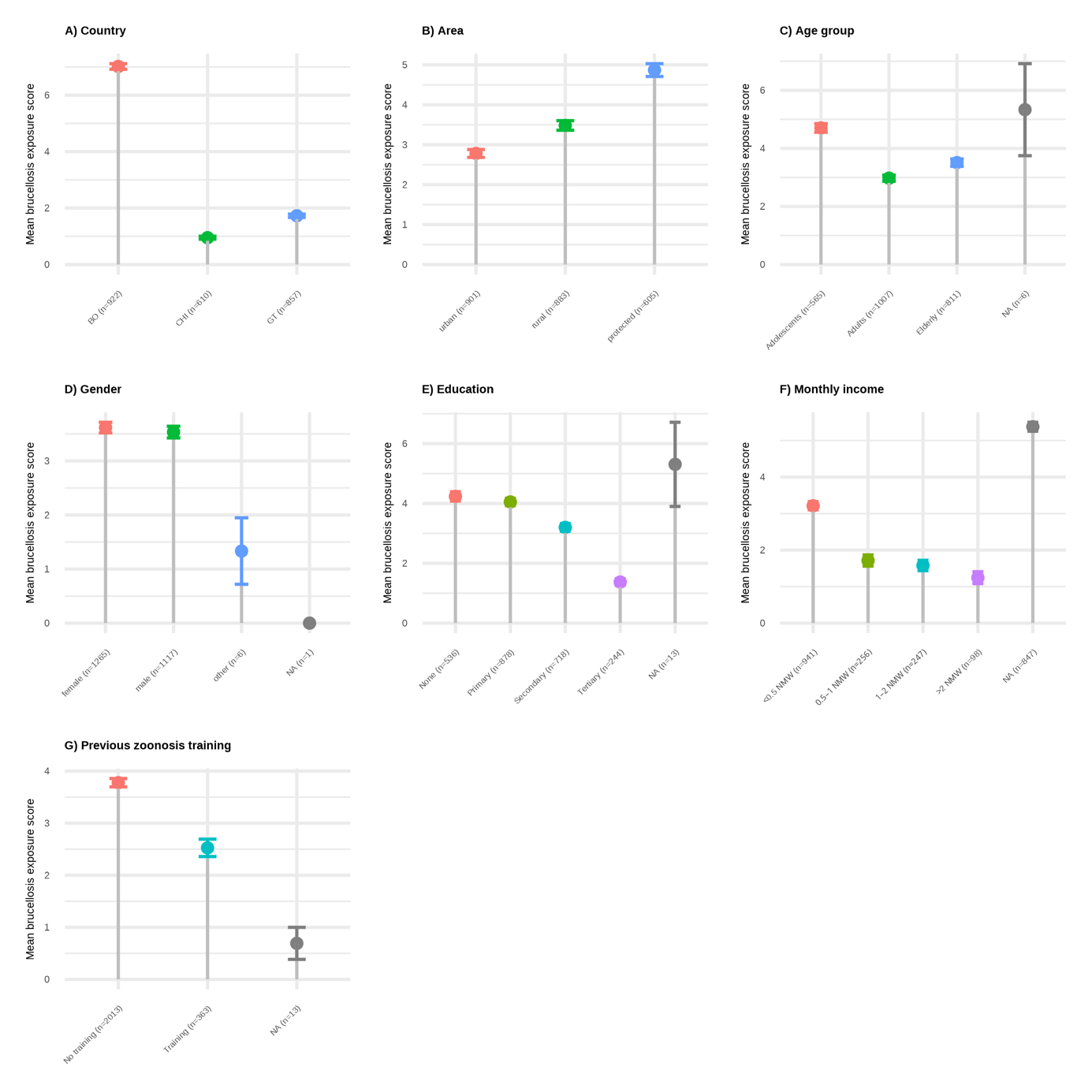


**Figure S5.** Mean brucellosis exposure scores across sociodemographic and knowledge-related factors for categories of (A) country, (B) area of residence, (C) age group, (D) gender, (E) education, (F) monthly income measured in National Minimum Wage (NMW), and (G) previous zoonoses training. Sample sizes per group are indicated on the x-axis.

**Table S6.** Association between sociodemographic and knowledge-related variables and brucellosis mean exposure score in multivariable linear regression.

| **Variable** | **Model Estimates (Beta)** | **95% CI^a^** | **p-Value^b^** |
| --- | --- | --- | --- |
| Country |  |  |  |
| Chile - Bolivia | -5.6 | -5.9, -5.2 | **<0.001** |
| Guatemala - Bolivia | -5.1 | -5.4, -4.7 | **<0.001** |
| Guatemala - Chile | 0.51 | 0.15, 0.87 | **0.002** |
| Age group |  |  |  |
| Adults - Adolescents | 0.06 | -0.39, 0.51 | 0.943 |
| Elderly - Adolescents | 0.21 | -0.26, 0.68 | 0.552 |
| Elderly - Adults | 0.15 | -0.15, 0.44 | 0.468 |
| Gender |  |  |  |
| male - female | 0.30 | 0.05, 0.55 | **0.013** |
| other - female | -0.50 | -2.9, 1.9 | 0.875 |
| other - male | -0.80 | -3.2, 1.6 | 0.708 |
| Education |  |  |  |
| Primary - None | 0.00 | -0.39, 0.39 | >0.999 |
| Secondary - None | -0.22 | -0.65, 0.21 | 0.559 |
| Secondary - Primary | -0.22 | -0.57, 0.14 | 0.400 |
| Tertiary - None | -0.58 | -1.1, -0.04 | **0.028** |
| Tertiary - Primary | -0.58 | -1.1, -0.10 | **0.011** |
| Tertiary - Secondary | -0.36 | -0.82, 0.09 | 0.170 |
| Monthly income^c^ |  |  |  |
| 0.5–1 NMW - <0.5 NMW | 0.00 | -0.39, 0.39 | >0.999 |
| 1–2 NMW - <0.5 NMW | -0.05 | -0.49, 0.39 | 0.992 |
| 1–2 NMW - 0.5–1 NMW | -0.05 | -0.54, 0.44 | 0.993 |
| >2 NMW - <0.5 NMW | -0.01 | -0.65, 0.63 | >0.999 |
| >2 NMW - 0.5–1 NMW | -0.01 | -0.68, 0.66 | >0.999 |
| >2 NMW - 1–2 NMW | 0.04 | -0.59, 0.67 | 0.998 |
| Previous training |  |  |  |
| Training - No training | -0.13 | -0.40, 0.14 | 0.341 |
| Area of Residence |  |  |  |
| rural - urban | 0.33 | 0.06, 0.60 | **0.010** |
| protected - urban | 0.06 | -0.31, 0.44 | 0.914 |
| protected - rural | -0.27 | -0.64, 0.11 | 0.219 |
| ^a^Confidence Interval; ^b^Significant effects are marked in bold (threshold : 0.05) ; ^c^Monthly income was categorized relative to the national minimum wage. | | | |


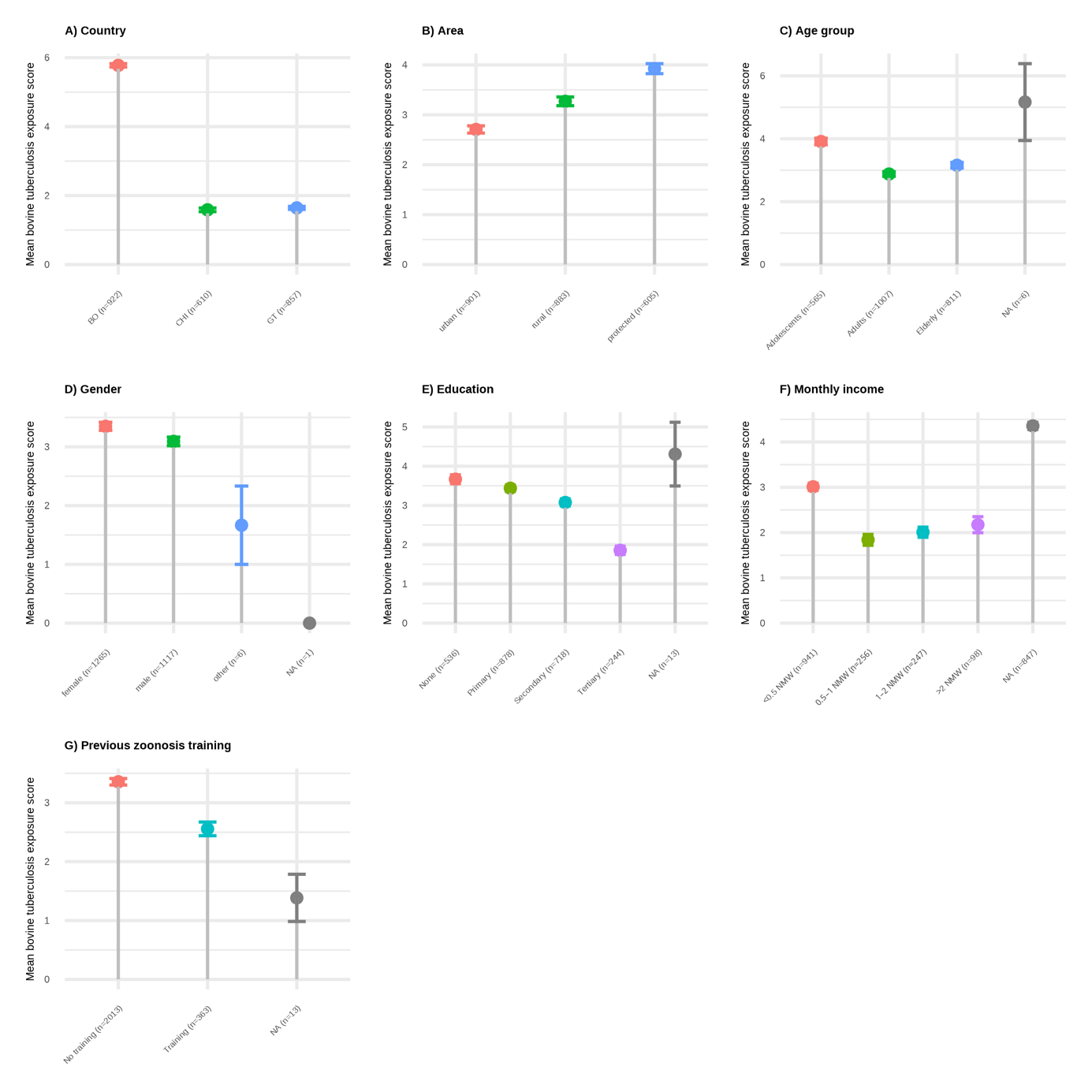


**Figure S7.** Mean bovine tuberculosis exposure scores across sociodemographic and knowledge-related factors for categories of (A) country, (B) area of residence, (C) age group, (D) gender, (E) education, (F) monthly income measured in National Minimum Wage (NMW), and (G) previous zoonoses training. Sample sizes per group are indicated on the x-axis.

**Table S8.** Association between sociodemographic and knowledge-related variables and bovine tuberculosis mean exposure score in multivariable linear regression.

| **Variable** | **Model Estimates (Beta)** | **95% CI^a^** | **p-Value^b^** |
| --- | --- | --- | --- |
| Country |  |  |  |
| Chile - Bolivia | -4.1 | -4.4, -3.9 | **<0.001** |
| Guatemala - Bolivia | -4.2 | -4.4, -3.9 | **<0.001** |
| Guatemala - Chile | -0.06 | -0.31, 0.19 | 0.859 |
| Age group |  |  |  |
| Adults - Adolescents | -0.01 | -0.33, 0.30 | 0.995 |
| Elderly - Adolescents | 0.02 | -0.32, 0.35 | 0.992 |
| Elderly - Adults | 0.03 | -0.18, 0.24 | 0.939 |
| Gender |  |  |  |
| male - female | 0.08 | -0.10, 0.25 | 0.548 |
| other - female | -0.36 | -2.0, 1.3 | 0.867 |
| other - male | -0.44 | -2.1, 1.2 | 0.810 |
| Education |  |  |  |
| Primary - None | -0.07 | -0.34, 0.21 | 0.927 |
| Secondary - None | -0.03 | -0.33, 0.27 | 0.992 |
| Secondary - Primary | 0.03 | -0.22, 0.28 | 0.988 |
| Tertiary - None | -0.35 | -0.73, 0.03 | 0.080 |
| Tertiary - Primary | -0.28 | -0.62, 0.05 | 0.133 |
| Tertiary - Secondary | -0.32 | -0.64, 0.00 | 0.054 |
| Monthly income^c^ |  |  |  |
| 0.5–1 NMW - <0.5 NMW | -0.12 | -0.39, 0.16 | 0.693 |
| 1–2 NMW - <0.5 NMW | -0.01 | -0.32, 0.30 | >0.999 |
| 1–2 NMW - 0.5–1 NMW | 0.10 | -0.24, 0.45 | 0.866 |
| >2 NMW - <0.5 NMW | 0.40 | -0.05, 0.85 | 0.108 |
| >2 NMW - 0.5–1 NMW | 0.51 | 0.04, 0.98 | **0.026** |
| >2 NMW - 1–2 NMW | 0.41 | -0.03, 0.85 | 0.083 |
| Previous training |  |  |  |
| Training - No training | 0.01 | -0.18, 0.20 | 0.919 |
| Area of Residence |  |  |  |
| rural - urban | 0.31 | 0.12, 0.49 | **<0.001** |
| protected - urban | 0.21 | -0.05, 0.47 | 0.150 |
| protected - rural | -0.10 | -0.36, 0.16 | 0.658 |
| ^a^Confidence Interval; ^b^Significant effects are marked in bold (threshold : 0.05) ; ^c^Monthly income was categorized relative to the national minimum wage. | | | |
